# Novel R tools for analysis of genome-wide population genetic data with emphasis on clonality

**DOI:** 10.3389/fgene.2015.00208

**Published:** 2015-06-10

**Authors:** Zhian N. Kamvar, Jonah C. Brooks, Niklaus J. Grünwald

**Affiliations:** ^1^Botany and Plant Pathology, Oregon State UniversityCorvallis, OR, USA; ^2^College of Electrical Engineering and Computer Science, Oregon State UniversityCorvallis, OR, USA; ^3^Horticultural Crops Research Laboratory, USDA Agricultural Research ServiceCorvallis, OR, USA

**Keywords:** clonality, population genomics, bootstrap, index of association, hierarchical analysis, sliding window

## Abstract

To gain a detailed understanding of how plant microbes evolve and adapt to hosts, pesticides, and other factors, knowledge of the population dynamics and evolutionary history of populations is crucial. Plant pathogen populations are often clonal or partially clonal which requires different analytical tools. With the advent of high throughput sequencing technologies, obtaining genome-wide population genetic data has become easier than ever before. We previously contributed the R package *poppr* specifically addressing issues with analysis of clonal populations. In this paper we provide several significant extensions to *poppr* with a focus on large, genome-wide SNP data. Specifically, we provide several new functionalities including the new function mlg.filter to define clone boundaries allowing for inspection and definition of what is a clonal lineage, minimum spanning networks with reticulation, a sliding-window analysis of the index of association, modular bootstrapping of any genetic distance, and analyses across any level of hierarchies.

## Introduction

To paraphrase Dobzhansky, nothing in the field of plant-microbe interactions makes sense except in the light of population genetics (Dobzhansky, 1973). Genetic forces such as selection and drift act on alleles in a population. Thus, a true understanding of how plant pathogens emerge, evolve and adapt to crops, fungicides, or other factors, can only be elucidated in the context of population level phenomena given the demographic history of populations (Milgroom et al., 1989; McDonald and Linde, 2002; Grünwald and Goss, 2011). The field of population genetics, in the era of whole genome resequencing, provides unprecedented power to describe the evolutionary history and population processes that drive coevolution between pathogens and hosts. This powerful field thus critically enables effective deployment of R genes, design of pathogen informed plant resistance breeding programs, and implementation of fungicide rotations that minimize emergence of resistance.

Most computational tools for population genetics are based on concepts developed for sexual model organisms. Populations that reproduce clonally or are polyploid are thus difficult to characterize using classical population genetic tools because theoretical assumptions underlying the theory are violated. Yet, many plant pathogen populations are at least partially clonal if not completely clonal (Anderson and Kohn, 1995; Milgroom, 1996). Thus, development of tools for analysis of clonal or polyploid populations is needed.

Genotyping by sequencing and whole genome resequencing provide the unprecedented ability to identify thousands of single nucleotide polymorphisms (SNPs) in populations (Luikart et al., 2003; Davey et al., 2011; Elshire et al., 2011). With traditional marker data (e.g., SSR, AFLP) a clone was typically defined as a unique multilocus genotype (MLG) (Falush et al., 2003; Taylor and Fisher, 2003; Grünwald and Hoheisel, 2006; Goss et al., 2009; Cooke et al., 2012). Availability of large SNP data sets provides new challenges for data analysis. These data are based on reduced representation libraries and high throughput sequencing with moderate sequencing depth which invariably results in substantial missing data, error in SNP calling due to sequencing error, lack of read depth or other sources of spurious allele calls (Mastretta-Yanes et al., 2015). It is thus not clear what a clone is in large SNP data sets and novel tools are required for definition of clone boundaries.

The research community using the R statistical and computing language (R Core Team, 2015) has developed a plethora of new resources for population genetic analysis. R is particularly appealing because all code is open source and functions can be evaluated and modified by any user. Recently, we introduced the R package *poppr* specifically developed for analysis of clonal populations (Kamvar et al., 2014b). *Poppr* previously introduced several novel features including the ability to conduct a hierarchical analysis across unlimited hierarchies, test for linkage association, graph minimum spanning networks or provide bootstrap support for Bruvo's distance in resulting trees. *Poppr* has been rapidly adopted and applied to a range of studies including for example horizontal transmission in leukemia of clams (Metzger et al., 2015), study of the vector-mediated parent-to-offspring transmission in an avian malaria-like parasite (Chakarov et al., 2015), and characterization of the emergence of the invasive forest pathogen *Hymenoscyphus pseudoalbidus* (Gross et al., 2014). It has also been used to implement real-time, online R based tools for visualizing relationships among unknown MLGs in reference databases (http://phytophthora-id.org/) (Grünwald et al., 2011).

Here, we introduce *poppr* 2.0, which provides a major update to *poppr* (Kamvar et al., 2014b) including novel tools for analysis of clonal populations specifically addressing large SNP data. Significant novel tools include functions for calculating clone boundaries and collapsing individuals into clonal groups based on a user-specified genetic distance threshold, sliding window analyses, genotype accumulation curves, reticulations in minimum spanning networks, and bootstrapping for any genetic distance.

## Implementations and examples

### Clonal identification

As highlighted in previous work, clone correction is an important component of population genetic analysis of organisms that are known to reproduce asexually (Milgroom, 1996; Grünwald et al., 2003; Kamvar et al., 2014b). This method is a partial correction for bias that affects metrics that rely on allele frequencies assuming panmixia and was initially designed for data with only a handful of markers. With the advent of large-scale sequencing and reduced- representation libraries, it has become easier to sequence tens of thousands of markers from hundreds of individuals (Davey and Blaxter, 2010; Davey et al., 2011; Elshire et al., 2011). With this larger number of markers, the genetic resolution is much greater, but the chance of genotyping error is also greatly increased and missing data is frequent (Mastretta-Yanes et al., 2015). Taking this fact and occasional somatic mutations into account, it would be impossible to separate true clones from independent individuals by just comparing what MLGs are different. We introduce a new method for collapsing unique multilocus genotypes determined by naive string comparison into multilocus lineages utilizing any genetic distance given three different clustering algorithms: farthest neighbor, nearest neighbor, and Unweighted Pair Group Method with Arithmetic Mean (UPGMA, average neighbor) (Sokal, 1958).

**Figure 1:**
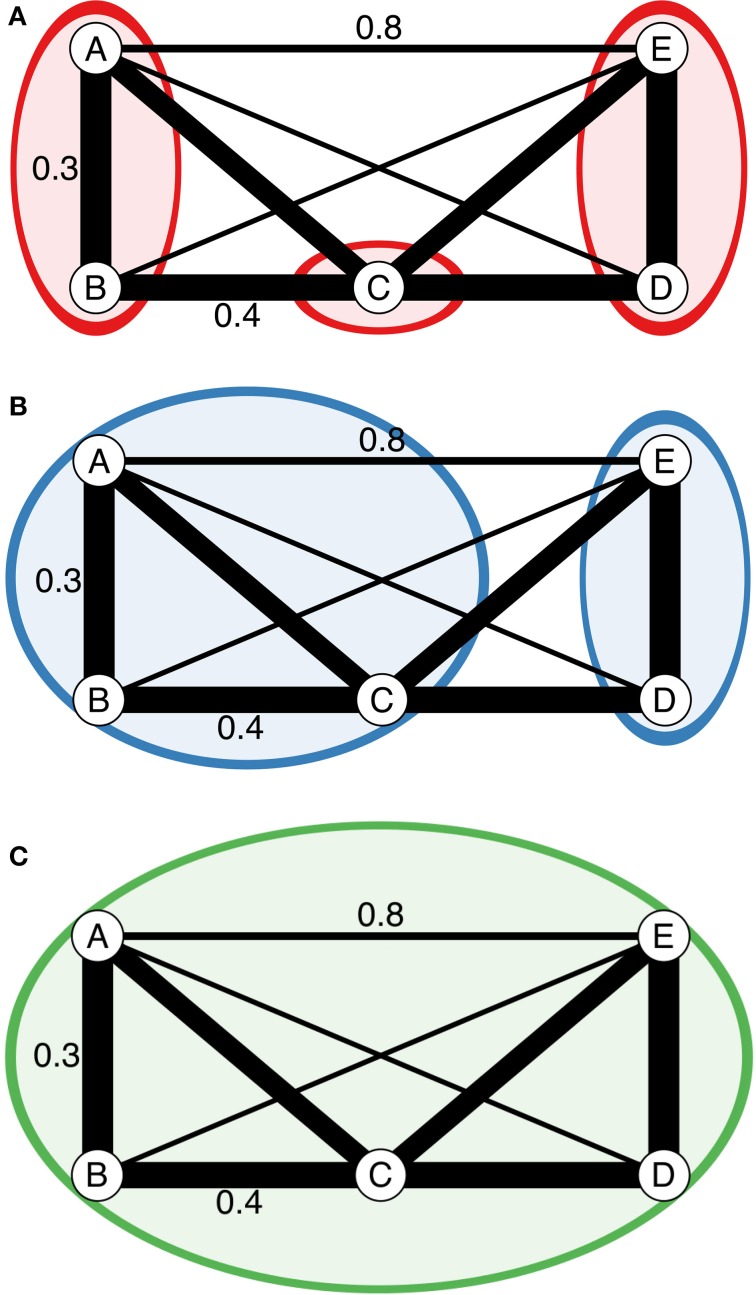
**Diagrammatic representation of the three clustering algorithms implemented in mlg.filter**. **(A–C**) Represent different clustering algorithms on the same imaginary network with a threshold of 0.451. Edge weights are represented in arbitrary units noted by the line thickness and numerical values next to the lines. All outer angles are 90°, so the un-labeled edge weights can be obtained with simple geometry. Colored circles represent clusters of genotypes. **(A)** Farthest neighbor clustering does not cluster nodes B and C because nodes A and C are more than a distance of 0.451 apart. **(B)** UPGMA (average neighbor) clustering clusters nodes A, B, and C together because the average distance between them and C is <0.451. **(C)** Nearest neighbor clustering clusters all nodes together because the minimum distance between them is always <0.451.

These clustering algorithms act on a distance matrix that is either provided by the user or generated via a function that will calculate a distance from genetic data such as bruvo.dist, which in particular applies to any level of ploidy (Bruvo et al., 2004). All algorithms have been implemented in C and utilize the OpenMP framework for optional parallel processing (Dagum and Menon, 1998). Default is the conservative farthest neighbor algorithm (Figure 1A), which will only cluster samples together if all samples in the cluster are at a distance less than the given threshold. By contrast, the nearest neighbor algorithm will have a chaining effect that will cluster samples akin to adding links on a chain where a sample can be included in a cluster if all of the samples have at least one connection below a given threshold (Figure 1C). The UPGMA, or average neighbor clustering algorithm is the one most familiar to biologists as it is often used to generate ultra-metric trees based on genetic distance (Figure 1B). This algorithm will cluster by creating a representative sample per cluster and joining clusters if these representative samples are closer than the given threshold.

We utilize data from the microbe *Phytophthora infestans* to show how the mlg.filter function collapses multilocus genotypes with Bruvo's distance assuming a genome addition model (Bruvo et al., 2004). *P. infestans* is the causal agent of potato late blight originating from Mexico that spread to Europe in the mid nineteenth century (Yoshida et al., 2013; Goss et al., 2014). *P. infestans* reproduces both clonally and sexually. The clonal lineages of *P. infestans* have been formally defined into 18 separate clonal lineages using a combination of various molecular methods including AFLP and microsatellite markers (Lees et al., 2006; Li et al., 2013). For these data, we used mlg.filter to detect all of the distance thresholds at which 18 multilocus lineages would be resolved. We used these thresholds to define multilocus lineages and create contingency tables and dendrograms to determine how well the multilocus lineages were detected.

**Figure 2:**
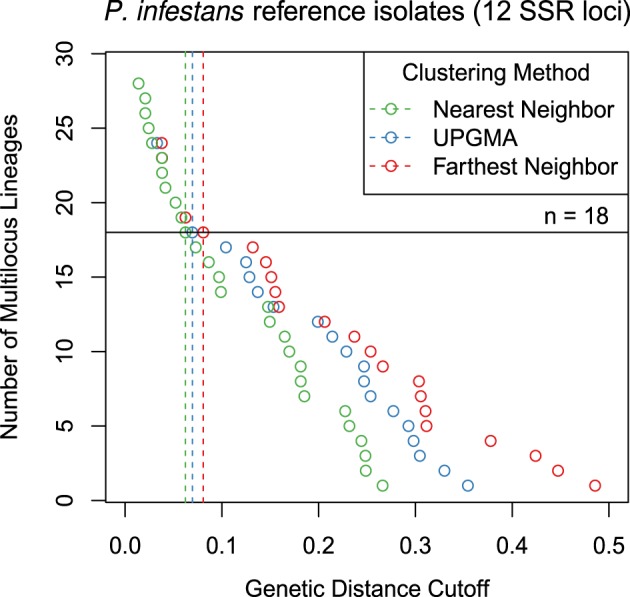
**Graphical representation of three different clustering algorithms collapsing multilocus genotypes for 12 SSR loci from** ***Phytophthora infestans*** **representing 18 clonal lineages**. The horizontal axis is Bruvo's genetic distance assuming the genome addition model. The vertical axis represents the number of multilocus lineages observed. Each point shows the threshold at which one would observe a given number of multilocus genotypes. The horizontal black line represents 18 multilocus genotypes and vertical dashed lines mark the thresholds used to collapse the multilocus genotypes into 18 multilocus lineages.

For the *P. infestans* population, the three algorithms were able to detect 18 multilocus lineages at different distance thresholds (Figure 2). Contingency tables between the described multilocus genotypes and the genotypes defined by distance show that most of the 18 lineages were resolved, except for US-8, which is polytomic (Table 1).

**Table 1:** **Contingency table comparing multilocus lineages (MLL) defined in Li et al. (2013) and Lees et al. (2006) (rows) to MLLs inferred from Bruvo's genetic distance (columns) at a threshold of 0.07 with the average neighbor algorithm (Sokal, 1958; Bruvo et al., 2004)**.

***P. infestans***	**Inferred MLL**																	
**MLL**	**3**	**4**	**5**	**6**	**8**	**10**	**12**	**15**	**16**	**17**	**18**	**20**	**21**	**22**	**24**	**25**	**27**	**28**
B	.	.	.	.	.	.	.	.	.	.	.	.	.	.	.	1	.	.
C	.	.	.	.	.	.	.	.	.	.	.	.	.	.	1	.	.	.
D.1	.	.	.	.	.	.	.	.	.	.	.	.	.	1	.	.	.	.
D.2	.	.	.	.	.	.	.	.	.	.	.	.	.	1	.	.	.	.
EU-13	.	.	.	.	.	.	.	.	1	.	.	.	.	.	.	.	.	.
EU-4	.	.	.	.	.	.	.	.	.	1	.	.	.	.	.	.	.	.
EU-5	.	.	.	.	.	.	.	.	.	.	2	.	.	.	.	.	.	.
EU-8	.	.	.	.	.	.	1	.	.	.	.	.	.	.	.	.	.	.
US-11	.	.	.	.	.	.	.	.	.	.	.	.	.	.	.	.	.	2
US-12	.	1	.	.	.	.	.	.	.	.	.	.	.	.	.	.	.	.
US-14	.	.	.	.	.	1	.	.	.	.	.	.	.	.	.	.	.	.
US-17	.	.	.	.	.	.	.	.	.	.	.	1	.	.	.	.	.	.
US-20	2	.	.	.	.	.	.	.	.	.	.	.	.	.	.	.	.	.
US-21	.	.	.	.	.	.	.	.	.	.	.	.	.	.	.	.	2	.
US-22	.	.	.	.	.	.	.	.	.	.	.	.	2	.	.	.	.	.
US-23	.	.	.	.	.	.	.	3	.	.	.	.	.	.	.	.	.	.
US-24	.	.	.	.	3	.	.	.	.	.	.	.	.	.	.	.	.	.
US-8	.	.	1	1	.	2	.	.	.	.	.	.	.	.	.	.	.	.

*Values in the table represent the number of times any given inferred MLL matches with a previously defined MLL. For example, in our original data set, there were three genotypes previously defined as the US-24 MLL. All three genotypes were also determined to cluster into a single MLL by filtering. In contrast, US-8 was determined to cluster into three different MLLs by filtering*.

We utilized simulated data to evaluate the effect of sequencing error and missing data on MLG calling. We constructed the data using the glSim function in *adegenet* (Jombart and Ahmed, 2011) to obtain a SNP data set for demonstration. Two diploid data sets were created, each with 10k SNPs (25% structured into two groups) and 200 samples with 10 ancestral populations of even sizes. Clones were created in one data set by marking each sample with a unique identifier and then randomly sampling with replacement. It is well documented that reduced- representation sequencing can introduce several erroneous calls and missing data (Mastretta-Yanes et al., 2015). To reflect this, we mutated SNPs at a rate of 10% and inserted an average of 10% missing data for each sample after clones were created, ensuring that no two sequences were alike. The number of mutations and missing data per sample were determined by sampling from a Poisson distribution with (λ = 1000). After pooling, 20% of the data set was randomly sampled for analysis. Genetic distance was obtained with the function bitwise.dist, which calculates the fraction of different sites between samples equivalent to Provesti's distance, counting missing data as equivalent in comparison (Prevosti et al., 1975).

All three filtering algorithms were run with a threshold of 1, returning a numeric vector of length n - 1 where each element represented a threshold at which two samples/clusters would join. Since each data set would have varying distances between samples, the clonal boundary threshold was defined as the midpoint of the largest gap between two thresholds that collapsed less than 50% of the data.

Out of the 100 simulations run, we found that across all methods, detection of duplicated samples had ~98% true positive fraction and ~0.8% false positive fraction indicating that this method is robust to simulated populations (Supplementary Materials^1^).

### Minimum spanning networks with reticulation

In its original iteration, *poppr* introduced minimum spanning networks that were based on the *igraph* function minimum.spanning.tree (Csardi and Nepusz, 2006). This algorithm produces a minimum spanning tree with no reticulations where nodes represent individual MLGs. In other minimum spanning network programs, reticulation is obtained by calculating the minimum spanning tree several times and returning the set of all edges included in the trees. Due to the way *igraph* has implemented Prim's algorithm, it is not possible to utilize this strategy, thus we implemented an internal C function to walk the space of minimum spanning trees based on genetic distance to connect groups of nodes with edges of equal weight.

To demonstrate the utility of minimum spanning networks with reticulation, we used two clonal data sets: the H3N2 flu virus data from the *adegenet* package using years of each epidemic as the population factor, and *Phytophthora ramorum* data from Nurseries and Oregon forests (Jombart et al., 2010; Kamvar et al., 2014a). Minimum spanning networks were created with and without reticulation using the *poppr* functions diss.dist and bruvo.msn for the H3N2 and *P. ramorum* data, respectively (Bruvo et al., 2004; Kamvar et al., 2014b). To detect mlg clusters, the infoMAP community detection algorithm was applied with 10,000 trials as implemented in the R package *igraph* version 0.7.1 utilizing genetic distance as edge weights and number of samples in each MLG as vertex weights (Csardi and Nepusz, 2006; Rosvall and Bergstrom, 2008).

To evaluate the results, we compared the number, size, and entropy (*H*) of the resulting communities as we expect a highly clonal organism with low genetic diversity to result in a few, large communities. We also created contingency tables of the community assignments with the defined populations and used those to calculate entropy using Shannon's index with the function diversity from the R package *vegan* version 2.2-1 (Shannon, 2001; Oksanen et al., 2015). A low entropy indicates presence of a few large communities whereas high entropy indicates presence of many small communities.

**Figure 3:**
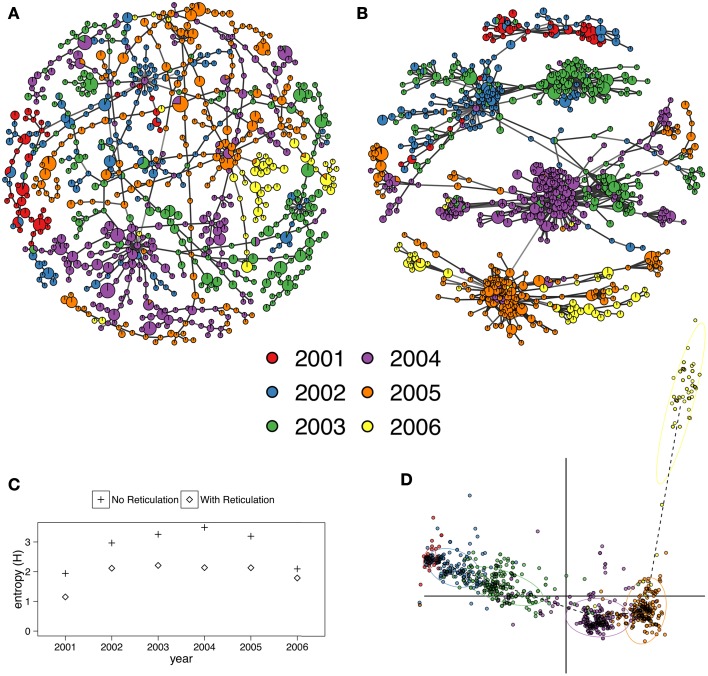
**(A,B)** Minimum spanning networks of the hemagglutinin (HA) segment of H3N2 viral DNA from the *adegenet* package representing flu epidemics from 2001 to 2006 without reticulation **(A)** and with reticulation **(B)** (Jombart, 2008; Jombart et al., 2010). Each node represents a unique multilocus genotype, colors represent epidemic year, and edge color represents absolute genetic distance. **(C)** Shannon entropy values for population assignments compared with communities determined by the infoMAP algorithm on **(A,B)**. **(D)** Graphic reproduced from Jombart et al. (2010) showing that the 2006 epidemic does not cluster neatly with the other years via Discriminant Analysis of Principal Components. Horizontal axis represents the first discriminant component. Vertical axis represents the second discriminant component.

The infoMAP algorithm revealed 63 communities with a maximum community size of 77 and *H* = 3.56) for the reticulate network of the H3N2 data and 117 communities with a maximum community size of 26 and *H* = 4.65 for the minimum spanning tree. The entropy across years was greatly decreased for all populations with the reticulate network compared to the minimum spanning tree (Figure 3). Note that the reticulated network (Figure 3B) showed patterns corresponding with those resulting from a discriminant analysis of principal components (Figure 3D) (Jombart et al., 2010).

Graph walking of the reticulated minimum spanning network of *P. ramorum* by the infoMAP algorithm revealed 16 communities with a maximum community size of 13 and *H* = 2.60. The un-reticulated minimum spanning tree revealed 20 communities with a maximum community size of 7 and *H* = 2.96. In the ability to predict Hunter Creek as belonging to a single community, the reticulated network was successful whereas the minimum spanning tree separated one genotype from that community. The entropy for the reticulated network was lower for all populations except for the coast population (Supplementary Materials^2^).

### Bootstrapping

Assessing population differentiation through methods such as *G_st_*, AMOVA, and Mantel tests relies on comparing samples within and across populations (Mantel, 1967; Nei, 1973; Excoffier et al., 1992). Confidence in distance metrics is related to the confidence in the markers to accurately represent the diversity of the data. Especially true with microsatellite markers, a single hyper-diverse locus can make a population appear to have more diversity based on genetic distance. Using a bootstrapping procedure of randomly sampling loci with replacement when calculating a distance matrix provides support for clades in hierarchical clustering.

Data in genind and genpop objects are represented as matrices with individuals in rows and alleles in columns (Jombart, 2008). This gives the advantage of being able to use R's matrix algebra capabilities to efficiently calculate genetic distance. Unfortunately, this also means that bootstrapping is a non- trivial task as all alleles at a single locus need to be sampled together. To remedy this, we have created an internal S4 class called “bootgen,” which extends the internal “gen” class from *adegenet*. This class can be created from any genind, genclone, or genpop object, and allows loci to be sampled with replacement. To further facilitate bootstrapping, a function called aboot, which stands for “any boot,” is introduced that will bootstrap any genclone, genind, or genpop object with any genetic distance that can be calculated from it.

**Figure 4:**
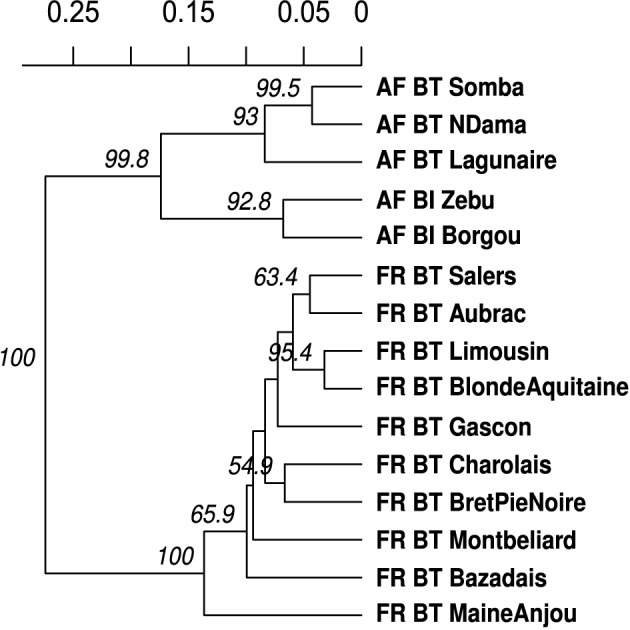
**UPGMA dendrogram generated from Nei's genetic distance on 15 breeds of** ***Bos taurus*** **(BT) or** ***Bos indicus*** **(BI) from Africa (AF) or France (FR)**. These data are from Laloë et al. (2007). Node labels represent bootstrap support (>50%) out of 1000 bootstrap replicates.

To demonstrate calculating a dendrogram with bootstrap support, we used the *poppr* function aboot on population allelic frequencies derived from the data set microbov in the *adegenet* package with 1000 bootstrap replicates (Laloë et al., 2007; Jombart, 2008). The resulting dendrogram shows bootstrap support values (>50%) (Figure 4) and used the following code:


library(“poppr”)
data(“microbov”, package = “adegenet”)
strata(microbov) <- data.frame(other(microbov))
setPop(microbov) <- ~coun/spe/breed
bov_pop <- genind2genpop(microbov)
set.seed(20150428)
pop_tree <- aboot(bov_pop, sample = 1000,
                           cutoff = 50)cutoff = 50)


### Genotype accumulation curve

Analysis of population genetics of clonal organisms often borrows from ecological methods such as analysis of diversity within populations (Milgroom, 1996; Grünwald et al., 2003; Arnaud-Hanod et al., 2007). When choosing markers for analysis, it is important to make sure that the observed diversity in your sample will not appreciably increase if an additional marker is added (Arnaud-Hanod et al., 2007). This concept is analogous to a species accumulation curve, obtained by rarefaction. The genotype accumulation curve in *poppr* is implemented in the function genotype_curve. The curve is constructed by randomly sampling *x* loci and counting the number of observed MLGs. This repeated *r* times for 1 locus up to *n-1* loci, creating *n-1* distributions of observed MLGs.

**Figure 5:**
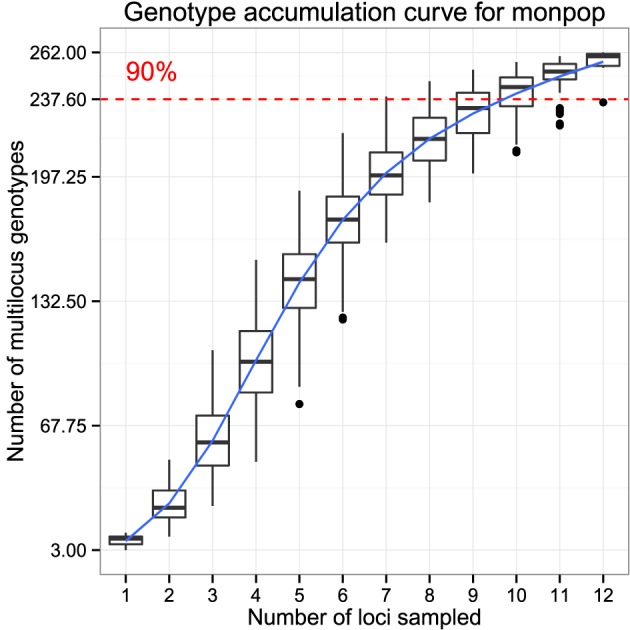
**Genotype accumulation curve for 694 isolates of the peach brown rot pathogen,** ***Monilinia fructicola*** **genotyped over 13 loci from Everhart and Scherm (2015)**. The horizontal axis represents the number of loci randomly sampled without replacement up to (*n* − 1) loci, the vertical axis shows the number of multilocus genotypes observed, up to 262, the number of unique multilocus genotypes in the data set. The red dashed line represents 90% of the total observed multilocus genotypes. A trendline (blue) has been added using the *ggplot2* function stat_smooth.


The following code example demonstrates the genotype accumulation curve for data from Everhart and Scherm (2015) showing that these data reach a small plateau and have a greatly decreased variance with 12 markers, indicating that there are enough markers such that adding more markers to the analysis will not create very many new genotypes (Figure 5).

library(“poppr”)
library(“ggplot2”)
data(“monpop", package = “poppr”)
set.seed(20150428)
genotype_curve(monpop, sample = 1000)
# get the last plot
p <- last_plot() + theme_bw()
# plot with a trendline
p + geom_smooth(aes(group = 1))


### Index of association

The index of association (*I_A_*) is a measure of multilocus linkage disequilibrium that is most often used to detect clonal reproduction within organisms that have the ability to reproduce via sexual or asexual processes (Brown et al., 1980; Smith et al., 1993). It was standardized in 2001 as ($\bar{r}$_*d*_) by Agapow and Burt (2001) to address the issue of scaling with increasing number of loci. This metric is typically applied to traditional dominant and co-dominant markers such as AFLPs, SNPs, or microsatellite markers. With the advent of high throughput sequencing, SNP data is now available in a genome-wide context and in very large matrices including thousands of SNPs. For this reason, we devised two approaches using the index of association for large numbers of markers typical for population genomic studies. Both functions utilize *adegenet*'s “genlight” object class, which efficiently stores 8 binary alleles in a single byte (Jombart and Ahmed, 2011). As calculation of the $\bar{r}$_*d*_ requires distance matrices of absolute number of differences, we utilize a function that calculates these distances directly from the compressed data called bitwise.dist.

The first approach is a sliding window analysis implemented in the function win.ia. It utilizes the position of markers in the genome to calculate $\bar{r}$_*d*_ among any number of SNPs found within a user-specified windowed region. It is important that this calculation utilize $\bar{r}$_*d*_ as the number of loci will be different within each window (Agapow and Burt, 2001). This approach would be suited for a quick calculation of linkage disequilibrium across the genome that can detect potential hotspots of LD that could be investigated further with more computationally intensive methods assuming that the number of samples << the number of loci.

As it would necessarily focus on loci within a short section of the genome that may or may not be recombining, a sliding window approach would not be good for utilizing $\bar{r}$_*d*_ as a test for clonal reproduction. A remedy for this is implemented in the function samp.ia, which will randomly sample *m* loci, calculate $\bar{r}$_*d*_, and repeat *r* times, thus creating a distribution of expected values of $\bar{r}$_*d*_.

**Figure 6:**
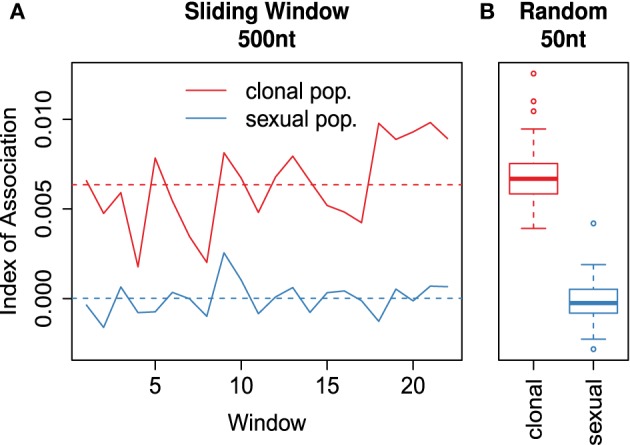
**(A)** Sliding window analysis of the standardized index of association ($\bar{r}$_*d*_) across a simulated 1.1 × 10^4^ nt chromosome containing 1100 variants among 100 individuals. Each window analyzed variants within 500nt chunks. The red line refers to the clonal and the blue line to the sexual populations. **(B)** boxplots showing 100 random samples of 50 variants to calculate a distribution of $\bar{r}$_*d*_ for the clonal (red) and sexual (blue) populations. Each box is centered around the mean, with whiskers extending out to 1.5 times the interquartile range. The median is indicated by the center line. **(A,B)** are plotted on the same y-axis.

To demonstrate the sliding window and random sampling of $\bar{r}$_*d*_ with respect to clonal populations, we simulated two populations containing 1100 neutral SNPs for 100 diploid individuals under the same initial seed. One population had individuals randomly sampled with replacement, representing the clonal population. After sampling, both populations had 5% random error and 1% missing data independently propagated across all samples. On average, we obtained a higher value of $\bar{r}$_*d*_ for the clonal population compared to the sexual population for both methods (Figure 6).

### Data format updates: population strata and hierarchies

Assessments of population structure through methods such as hierarchical *F_st_* (Goudet, 2005) and AMOVA (Michalakis and Excoffier, 1996) require hierarchical sampling of populations across space or time (Linde et al., 2002; Grünwald and Hoheisel, 2006; Everhart and Scherm, 2015). With clonal organisms, basic practice has been to clone-censor data to avoid downward bias in diversity due to duplicated genotypes that may or may not represent different samples (Milgroom, 1996). This correction should be performed with respect to a population hierarchy to accurately reflect the biology of the organism. Traditional data structures for population genetic data in most analysis tools allow for only one level of hierarchical definition. The investigator thus had to provide the data set for analysis at each hierarchical level.

To facilitate handling hierarchical and mutlilocus genotypic metadata, *poppr* version 1.1 introduced a new S4 data object called “genclone,” extending *adegenet*'s “genind” object (Kamvar and Grünwald, unpublished). The genclone object formalized the definitions of multilocus genotypes and population hierarchies by adding two slots called “mlg” and “hierarchy” that carried a numeric vector and a data frame, respectively. These new slots allow for increased efficiency and ease of use by allowing these metadata to travel with the genetic data. The hierarchy slot in particular contains a data frame where each column represents a separate hierarchical level. This is then used to set the population factor of the data by supplying a hierarchical formula containing one or more column names of the data frame in the hierarchy slot.

The functionality represented by the hierarchy slot has now been migrated from the *poppr* to the *adegenet* package version 2.0 to allow hierarchical analysis in *adegenet, poppr*, and other dependent packages. The prior *poppr* hierarchy slot and methods have now been renamed strata in *adegenet*. A short example of the utility of these methods can be seen in the code segment under Bootstrapping, above. This migration provides end users with a broader ability to analyze data hierarchically in R across packages.

## Availability

As of this writing, the *poppr* R package version 2.0 containing all of the features described here is located at https://github.com/grunwaldlab/poppr/tree/2.0-rc. It is necessary to install *adegenet* 2.0 before installing *poppr*. It can be found at https://github.com/thibautjombart/adegenet. Both of these can be installed via the R package *devtools* (Wickham and Chang, 2015). More information and example code can be found in the Supplementary Materials^3^.

### Requirements

- R version 3.0 or better.
- A C compiler. For windows, it can be obtained via Rtools (http://cran.r-project.org/bin/windows/Rtools/). On OSX, it can be obtained via Xcode. For parallel support, gcc version 4.6 or better is needed.

### Installation

From within R, *poppr* can be installed via:


install.packages(“devtools”)
library(“devtools”)
install_github(“thibautjombart/adegenet”)
install_github(“grunwaldlab/poppr@2.0-rc”)


Several population genetics packages in R are currently going through a major upgrade following the 2015 R hackathon on population genetics (https://github.com/NESCent/r-popgen-hackathon) and have not yet been updated in CRAN. We will upload *poppr* 2.0 to CRAN once all other reverse dependent packages have been updated.

## Discussion

Given low cost and high throughput of current sequencing technologies we are entering a new era of population genetics where large SNP data sets with thousands of markers are becoming available for large populations in a genome- wide context. This data provides new possibilities and challenges for population genetic analyses. We provide novel tools that enable analysis of this data in R with a particular emphasis on clonal organisms.

Particularly useful is the implementation of $\bar{r}$_*d*_ in a genomic context (Agapow and Burt, 2001). Random sampling of loci across the genome can give an expected distribution of $\bar{r}$_*d*_, which is expected to have a mean of zero for panmictic populations. This metric is not affected by the number of loci sampled, is model free, and has the ability to detect population structure. $\bar{r}$_*d*_ is also implemented for sliding window analyses that are useful to detect candidate regions of linkage disequilibrium for further analysis.

Clustering multilocus genotypes into multilocus lineages based on genetic distances is a non-trivial task given large SNP data sets. Moreover, this has not previously been implemented for genomic data for clonal populations. Clonal assignment has previously been available in the programs GenClone and Genodive for classical markers (Meirmans and Van Tienderen, 2004; Arnaud-Hanod et al., 2007). Our method with mlg.filter builds upon this idea and allows the user to choose between three different approaches for clustering MLGs. The choice of clustering algorithm has an impact on the data (Figures 1, 2), where for example a genetic distance cutoff of 0.1 would be the difference between 14 multilocus lineages (MLLs) and 17 MLLs for nearest neighbor and UPGMA clustering, respectively (Figure 2). The option to choose the clustering algorithm gives the user the ability to choose what is biologically relevant to their populations. While there is not one optimal procedure for defining boundaries in clonal lineages, our tool provides a means of exploring the potential MLG or MLL boundary space.

Minimum spanning networks are a useful tool to analyze the relationships between individuals in a population, because it reduces the complexity of a distance matrix to the connections that are strongest. By default, these networks are drawn without reticulations, but for clonal organisms where many of the connections between samples are equivalent, the minimum spanning network appears as a chain and reduces the information that can be communicated. This is problematic because the ability to detect population structure with one instance of a minimum spanning network is limited. Adding reticulation into the minimum spanning network thus presents all equivalent connections and allows population structure to be more readily detectable. As shown in Figure 3, population structure is apparent both visually and by graph community detection algorithms such as the infoMAP algorithm (Rosvall and Bergstrom, 2008). Additionally, the current implementation in *poppr* has been successfully used in analyses such as reconstruction of the *P. ramorum* epidemic in Oregon forests (Kamvar et al., 2014a, 2015).

*Poppr* 2.0 is open source and available on GitHub. Members of the community are invited to contribute by raising issues or pull requests on our repository at https://github.com/grunwaldlab/poppr/issues.

## Author contributions

ZK and JB wrote and tested the code. ZK maintains the code. ZK and NG conceived, discussed implications, and wrote the manuscript. NG coordinated the collaborative effort.

### Conflict of interest statement

The authors declare that the research was conducted in the absence of any commercial or financial relationships that could be construed as a potential conflict of interest.

## Footnotes

^1^Supplementary data available at https://github.com/grunwaldlab/supplementary-poppr-2.0.

^2^Supplementary data available at https://github.com/grunwaldlab/supplementary-poppr-2.0.

^3^Supplementary data available at https://github.com/grunwaldlab/supplementary-poppr-2.0.
